# Efficacy of a personalised alcohol approach bias modification smartphone app in people accessing outpatient alcohol use disorder treatment: A randomised controlled trial

**DOI:** 10.1111/add.70184

**Published:** 2025-09-04

**Authors:** Joshua B. B. Garfield, Bosco Rowland, Samuel K. Liu, Hugh Piercy, Yvonne Bonomo, Danielle Whelan, Victoria Manning

**Affiliations:** ^1^ Monash Addiction Research Centre, Eastern Health Clinical School Monash University Melbourne Australia; ^2^ Turning Point Eastern Health Richmond Victoria Australia; ^3^ Faculty of Health, School of Psychology Deakin University Burwood Victoria Australia; ^4^ Department of Addiction Medicine St Vincent's Hospital Melbourne Melbourne Australia; ^5^ Women's Alcohol and Drug Service The Royal Women's Hospital Melbourne Australia; ^6^ Department of Medicine The University of Melbourne Melbourne Australia

**Keywords:** addiction, alcohol use disorder, approach bias modification, cognitive bias modification, outpatient treatment, randomised controlled trial, smartphone app

## Abstract

**Background and aims:**

Several randomised controlled trials (RCTs) have demonstrated that delivering approach bias modification (ApBM) during residential alcohol use disorder (AUD) treatment helps prevent post‐treatment relapse. However, few studies have examined ApBM's efficacy for AUD in outpatients. We trialled a personalised ApBM smartphone app in individuals receiving outpatient AUD treatment.

**Design:**

This double‐blind RCT randomised participants to receive ApBM or sham training, adjunctive to treatment as usual.

**Setting:**

Participants were recruited from alcohol and other drug treatment services in Melbourne, Australia.

**Participants:**

79 participants (mean age 46.6 years; 45 male, 34 female) installed the app between May 2022 and January 2024.

**Intervention and comparator:**

In the ApBM condition, the app delivered personalised, gamified ApBM. Notifications prompted participants (*n* = 39) to complete 2 ApBM sessions weekly for 4 weeks. The control version prompted participants (*n* = 40) to complete a weekly sham‐training task for 4 weeks.

**Measurements:**

The primary outcome was number of standard drinks (10 g pure alcohol) consumed in week 4 of the intervention period, self‐reported in the app. Secondary outcomes included past‐week standard drinks at 8‐week and 16‐week follow‐ups, past‐week drinking days, past‐week heavy drinking days (HDDs; days when ≥5 standard drinks were consumed) and questionnaire measures of AUD severity, quality of life and alcohol craving. Primary analyses followed an intention‐to‐treat (ITT) approach, with secondary complete‐case sensitivity analyses also conducted for all outcomes.

**Findings:**

Groups did not statistically significantly differ in the primary outcome [values from ITT negative binomial model: ApBM = 75.49 standard drinks, control = 71.34 standard drinks, difference = 4.16, 95% confidence interval (CI) = −42.37 to 50.69, *P* = 0.859]. Most analyses of secondary outcomes showed statistically non‐significant effects, with the only exception being past‐week standard drinks at the 16‐week follow‐up, where ApBM participants showed statistically significantly larger decreases than controls in past‐week standard drinks (reduction of 14.6, relative to baseline, versus 2.1 in controls; interaction β = −12.53, 95% CI = −23.85 to −1.22; *P* = 0.030). Time x group interaction effects were statistically non‐significant for all other secondary outcomes (*P*s > 0.069).

**Conclusions:**

A smartphone app using approach bias modification showed no evidence for reducing alcohol use among alcohol use disorder outpatients after 4 weeks, or evidence for effects on most secondary outcomes, although 16‐week follow‐up results suggested that approach bias modification may have facilitated delayed/longer‐term reductions in alcohol use.

## INTRODUCTION

Approach bias modification (ApBM) is an intervention for alcohol use disorder (AUD) designed to reduce alcohol approach bias (the automatic tendency to approach alcohol‐related stimuli) [1]. ApBM involves practicing repeatedly ‘avoiding’ alcohol‐related images, while ‘approaching’ non‐alcohol‐related images. In most trials that have demonstrated ApBM's efficacy, participants responded to images presented on a computer monitor by ‘pushing’ or ‘pulling’ a joystick, causing images to shrink or expand, respectively, to simulate ‘avoidance’ and ‘approach’ [2, 3, 4, 5, 6, 7, 8, 9]. Several studies testing this method during residential AUD treatment have demonstrated that ApBM reduces approach bias [2, 4, 6, 9] and likelihood of post‐treatment relapse [2, 4, 5, 6, 7, 8, 9]. However, research examining ApBM's efficacy in other treatment settings has been limited.

Because outpatient treatment of AUD is more common than residential treatment [10], establishing the effectiveness of ApBM in outpatient settings could (if successful) benefit more patients. However, delivering ApBM in‐person, using a computer and joystick, may be impractical in outpatient settings. Trials supporting ApBM's efficacy delivered multiple training sessions per week, and outpatient appointments are not typically frequent enough to allow such frequent training. Moreover, the increase in remote delivery of outpatient addiction treatment via telehealth means some clients may not attend in‐person treatment at all.

The only randomised controlled trial (RCT) to examine ApBM's efficacy in AUD outpatients to date addressed these limitations by delivering ApBM online. Participants completed training sessions on their home computer, pressing designated keyboard keys to cause images to shrink or expand [11]. This RCT found that eight ApBM sessions reduced approach bias, but did not significantly reduce alcohol consumption, relative to sham‐training controls, although the authors noted the study was likely underpowered [11]. Moreover, this mode of delivery is still limited because it requires clients to have a home computer, which may be unrealistic for some clients.

Delivery of ApBM via a smartphone app may extend its reach and also enable clients to practice ApBM at convenient times for them and/or in contexts where they experience elevated cravings or risk of relapse. There have been few trials of alcohol ApBM smartphone apps to date. Crane *et al*. [12] reported that participants who received an app that delivered a combination of ApBM and normative feedback reduced their weekly alcohol consumption significantly more, over a 28‐day period, than participants who received an app that lacked the combination of these two interventions. However, a later re‐analysis of their outcomes in a larger sample found a non‐significant effect [13]. Laurens *et al*. [14] reported that participants' alcohol consumption reduced after 3 weeks of using an app that delivered two ApBM sessions per week, but this study lacked a control group. Peerenboom *et al*. [15] found that completing an ApBM session on a smartphone resulted in lower alcohol craving and Alcohol Use Disorder Identification Test (AUDIT) scores 1 week later, relative to sham‐training controls. However, their app delivered only a single, unusually long ApBM session (800 image presentations), an approach that is unlikely to be implemented in practice. Moreover, they did not assess outcomes beyond 1 week post‐training. Importantly, these studies all recruited people from the general community who were drinking heavily, not people in treatment, hence the applicability of their findings to clinical populations is unclear.

We previously developed an alcohol ApBM smartphone app, called ‘SWiPE’ [16]. SWiPE differed from previous ApBM programs by allowing users to personalise the stimuli used in training. Because approach bias is hypothesised to arise from repeated alcohol‐reward associative conditioning experiences [17], it may be specific to stimuli related to the drinks an individual frequently consumes. Therefore, using personally relevant alcohol images in ApBM training may more effectively reduce approach bias and be more engaging, as previously suggested by others [14, 18]. SWiPE also allowed users to select images representing positive personal goals or personally preferred healthy sources of pleasure as the stimuli they were trained to approach, which is consistent with recommendations to align training with individuals' goals for behavioural change or offer alternative strategies to manage cravings [18, 19, 20, 21]. In an open‐label feasibility study of SWiPE, participants recruited from the general community who reported hazardous drinking (i.e. AUDIT score ≥8) showed significant reductions between baseline and post‐training assessments in past‐week alcohol consumption, cravings and dependence severity [22]. However, this study lacked a control group and the sample mostly (91%) comprised non‐treatment‐seeking individuals.

We subsequently redeveloped SWiPE for use in RCTs, enabling it to deliver both ApBM and sham‐training control conditions and improving its functionality based on qualitative feedback from SWiPE trial participants [23]. To preserve participant blinding after the publicity SWiPE received, we renamed the new version ‘AAT‐App’ (‘Alcohol Avoidance Training App’). The present trial aimed to determine whether using AAT‐App to provide ApBM to outpatient AUD treatment clients improved outcomes, relative to sham‐training controls. We hypothesised that participants in the ApBM condition would report significantly less alcohol use, craving and dependence severity; and better quality of life, relative to controls. Consistent with previous ApBM app studies that tested alcohol consumption outcomes at the end of 3 to 4 week interventions [12, 14, 22], the primary outcome was the number of standard drinks consumed in the final week of the 4‐week intervention.

## METHODS

### Design

This double‐blind RCT recruited clients receiving outpatient treatment for alcohol use problems from addiction treatment services in the Melbourne metropolitan region (Australia). We randomised them to receive the ApBM or sham‐training control version of AAT‐App (adjunctive to treatment as usual). Originally, recruitment was limited to participants referred from six participating treatment services. However, in July 2023, recruitment was opened to other services' clients. The primary outcome was measured at the end of the 4‐week intervention period. Post‐intervention follow‐ups were completed at weeks 8 and 16.

### Participants

Seventy‐nine participants were randomised between 26 May 2022 and 23 January 2024. Recruitment ended because of funding limitations. Follow‐ups concluded on 15 May 2024. Inclusion criteria were: age ≥18 years; owned an Android or Apple smartphone with an Australian phone number and recently updated operating system; accessing outpatient treatment for alcohol problems *; and AUDIT score ≥8. Exclusion criteria were: in residential rehabilitation within the past 4 weeks; past‐week inpatient treatment (e.g. hospital or residential withdrawal treatment); or planning to enter inpatient/residential treatment within the next month. We also excluded people undergoing intensive non‐residential rehabilitation programs.

### Intervention

#### ApBM condition

Details of the personalisation, gamification, task instructions and task training parameters are provided in the Supporting information. In short, before the first ApBM session, participants selected six alcohol and six positive images. Each session involved 156 image presentations (13 presentations of each of the 12 selected images). Participants were trained to avoid 92.3% of alcohol image presentations and approach 92.3% of positive image presentations. App notifications reminded participants to complete two training sessions each week during the 4‐week intervention period. The average session took 3.9 minutes (SD = 2.0).

#### Control condition

Details of sham training task instructions, training parameters and rationale for the control condition design are provided in the Supporting information. In short, each sham training session involved 80 presentations of alcohol‐related and neutral images. Images were not personalised (i.e. all control participants responded to the same images). Participants were trained to approach 50% and avoid 50% of both alcohol and neutral images. After completing their first session, participants were prompted to complete one task at the end of each of the following 4 weeks.

### Measures

#### Primary outcome

At the end of the 4‐week intervention period, the app asked participants to estimate how many standard drinks (i.e. a drink containing 10 g of pure ethanol) they consumed on each day of the past week. The app offered examples of standard drink equivalents for common beverages and included a link to an online resource [24] to help participants estimate their consumption. The number of drinks consumed were summed across all 7 days to derive the total number of standard drinks consumed in week 4.

#### Secondary outcomes

*Secondary alcohol use outcomes*. Alcohol use was measured at week 8 and 16 using the same method used at week 4. Other past‐week drinking indices derived from this measure at weeks 4, 8 and 16 included ‘drinking days’ (number of days when any alcohol was consumed); ‘heavy drinking days’ (HDDs) (number of days when ≥5 standard drinks were consumed); and abstinence (an entire week when no alcohol was consumed).

*Alcohol problem severity*. The AUDIT [25] was used to measure AUD severity. We modified its wording to enquire about the past 3 months (instead of the standard 1 year) to allow assessment of equivalent, non‐overlapping periods at baseline and 16‐week follow‐up. The Severity of Dependence Scale (SDS) [26] was used to measure past‐month severity of psychological dependence. Because the SDS was originally designed to measure heroin, cocaine and amphetamine dependence, we modified items' wording to improve their relevance to alcohol, similar to the wording validated by Gossop *et al*. [27].

*Alcohol craving*. The Craving Experience Questionnaire frequency scale (CEQ‐F) [28] assessed past‐week craving frequency.

*Quality of life and health*. Three items [each rated on a scale from 0 (poor) to 10 (good)] adapted from the Australian Treatment Outcomes Profile (ATOP) [29] assessed past‐month psychological health, physical health and overall quality of life.

#### Other measures

*Demographic and treatment information*. A survey hosted on Qualtrics Plus assessed participants' age, gender, race, whether English was their first language, education, employment status, the date they began their current episode of outpatient treatment, the type(s) of outpatient treatment they were receiving for alcohol use, their treatment goal (i.e. abstinence or moderation) and other drugs of concern they were receiving treatment for.

*Baseline alcohol use*. Alcohol use in the week before installing the app was measured in the same manner described above for weeks 4, 8 and 16.

*App ratings*. Participants completed the ‘functionality’ and ‘aesthetics’ sub‐scales and the ‘app subjective quality’ section of the user version of the Mobile Application Rating Scale (uMARS) [30]. Additionally, participants rated the statements: ‘The app helped me reduce my alcohol use’; ‘The app increased my alcohol consumption’; ‘The app helped reduce my cravings for alcohol’; and ‘The app increased my cravings for alcohol’, using a 5‐point Likert scale (1: strongly disagree; 5: strongly agree).

*Blinding success*. In the 16‐week follow‐up survey, participants were asked to guess whether they had received ‘active brain training that was designed to change automatic reactions to alcohol images’ or the ‘control version where the training task was not designed to change automatic reactions to alcohol’.

*Training completion*. Participants' records in the back‐end app database included the date and time of each training session, allowing quantification of the number of sessions completed.

### Procedure

A data manager not involved with data collection or analyses generated separate randomisation sequences for each of the six original participating services (i.e. randomisation was stratified by treatment service), using a 1:1 allocation ratio and block sizes ranging from 2 to 6. When recruitment was later opened to clients of other services, a seventh randomisation sequence was generated for participants from any service other than the six original sites.

Clinicians at participating services were provided with eligibility criteria and were asked to inform potentially eligible clients of the trial and refer them to the researchers if they were interested. At some services, posters displayed in waiting areas or notices placed on the service's website allowed clients to refer themselves to the researchers. Researchers phoned referred clients to explain the study and, if the client was interested, administered the AUDIT and other questions to confirm eligibility. If the client was eligible, the researcher sent them a link to a Qualtrics form that displayed detailed participant information and asked them to confirm their consent to participate. If they consented, the Qualtrics survey collected demographics, SDS, CEQ‐F and ATOP data.

Once a participant completed the baseline survey, researchers sent them an SMS containing an app download link and a unique access code (selecting the first unused code from the appropriate randomisation list), which activated either the ApBM or control version of the app. Researchers who distributed the access codes were blinded to the randomisation sequence, and to which codes activated which conditions, until completion of data collection. After completing the alcohol consumption questions at the end of week 4, an in‐app link directed participants to the 4‐week follow‐up Qualtrics survey (including SDS, CEQ‐F, ATOP, uMARS and app ratings). This link expired after 7 days.

Twenty‐eight days after the end of week 4, an app notification prompted participants to report their past‐week alcohol consumption and then follow a link to the 8‐week follow‐up Qualtrics survey (SDS, CEQ‐F and ATOP), which expired after 14 days. Another 56 days later, a final app notification prompted participants to complete a 16‐week follow‐up comprising in‐app alcohol use questions and a Qualtrics survey including SDS, CEQ‐F, ATOP items, AUDIT and asking them to guess which condition they had been allocated to. The 16‐week follow‐up Qualtrics survey expired after 28 days. Participants received a $20 (Australian dollars) gift voucher for completing each follow‐up survey. The study was approved by the St Vincent's Hospital Melbourne Human Research Ethics Committee (HREC; 268/21) and the Monash University HREC (project number: 31260). The study was pre‐registered at clinicaltrials.gov (NCT05120856; registered 15 November 2021) and the trial protocol can be found at https://clinicaltrials.gov/ct2/show/NCT05120856.

### Statistical methods

Analyses were completed in R version 4.2.3 and STATA version 18. Statistical significance was ascertained using α = 0.05. Consistent with intention‐to‐treat (ITT) principles, all participants who installed the app were included in the analysis set.

#### Sample size and power

The original sample size calculation was not based on previous work, because no studies showing viable effects of personalised ApBM apps in AUD outpatients were available when we planned this trial (2021). Instead, we estimated the sample size necessary to achieve 90% power to detect a global generic small effect size for a time × group interaction across the four repeated measures of past‐week drinking (GPower 3.1). This indicated that 178 participants (89 per group) would be necessary. However, despite trial extensions, only 79 participants had installed the app when funding ceased. A *post hoc* power analysis, using a negative binomial model in Stata 18, involving 1000 simulations, was conducted based on the observed means, sample size and dispersion parameter (0.3) at follow up. Assuming a small expected between group difference of two standard drinks at 4‐week follow‐up, the achieved power for the primary outcome was 14.2%.

#### Primary outcome

Based on inspection of the primary outcome's distribution, Poisson and negative binomial probability functions were considered. Overdispersion was examined by inspection of variance and the log dispersion parameter in the negative binomial model. Akaike information criterion and Bayesian information criterion fit statistics were also compared between Poisson and negative binomial models. Zero‐inflation was examined using abstinence (yes/no) as the inflation offset variable, and was not detected. A negative binomial model was selected because the log dispersion parameter was significantly different from zero. Regression analysis identified that baseline past‐week standard drinks predicted past‐week standard drinks at week 4 (β = 0.74; *P* < 0.001 in ITT analysis; β = 0.58; *P* < 0.001 in complete‐case analysis), therefore, baseline standard drinks was included as a covariate in this analysis. Based on our experience that AUD treatment clients tend to be less likely to complete follow‐ups if they have reverted to heavy alcohol consumption, missing data was assumed to be missing ‘not at random’ (MNAR). Hence, the primary (ITT) analysis carried forward the baseline value if a participant's week 4 value was missing. A secondary sensitivity analysis used only observed data (i.e. no imputation, not ITT), and hence, modelled a missing ‘completely at random’ (MCAR) assumption.

#### Secondary outcomes

Secondary outcomes were tested using models that included all four time points (baseline; weeks 4, 8 and 16). These models fitted fixed effects of group, time, their interaction and random effects for participants within groups, using maximum likelihood to calculate predicted means based on estimated effects, with time treated as a categorical variable. For secondary outcomes based on count data (standard drinks at week 8 and 16; drinking days; HDDs), Poisson and negative binomial probability functions were considered, as described for the primary outcome. None of these outcomes were identified as showing zero‐inflation. We selected a linear mixed‐effects model for past‐week standard drinks at later follow‐ups, and the Poisson function for drinking days and HDDs. For outcomes that used continuous scales (craving, SDS, AUDIT and ATOP items), linear mixed models were used. Abstinence was analysed using mixed effects logistic regression. Following the MNAR‐based approach described for the primary outcome, last‐outcome‐carried‐forward (LOCF) imputation was used for the ITT analyses of secondary outcomes. Sensitivity analyses with no imputation (i.e. non‐ITT, MCAR‐based) were also conducted.

Between‐group differences in uMARS and app ratings were analysed using Welch's *t* tests. We used Pearson's χ^2^ tests to compare proportions of participants who completed each follow‐up in each group. We also used a Pearson's χ^2^ test to compare the proportion of participants who guessed they received the ‘active training’ between groups. Analyses of uMARS, app ratings and condition guess used only observed data.

## RESULTS

**FIGURE 1:**
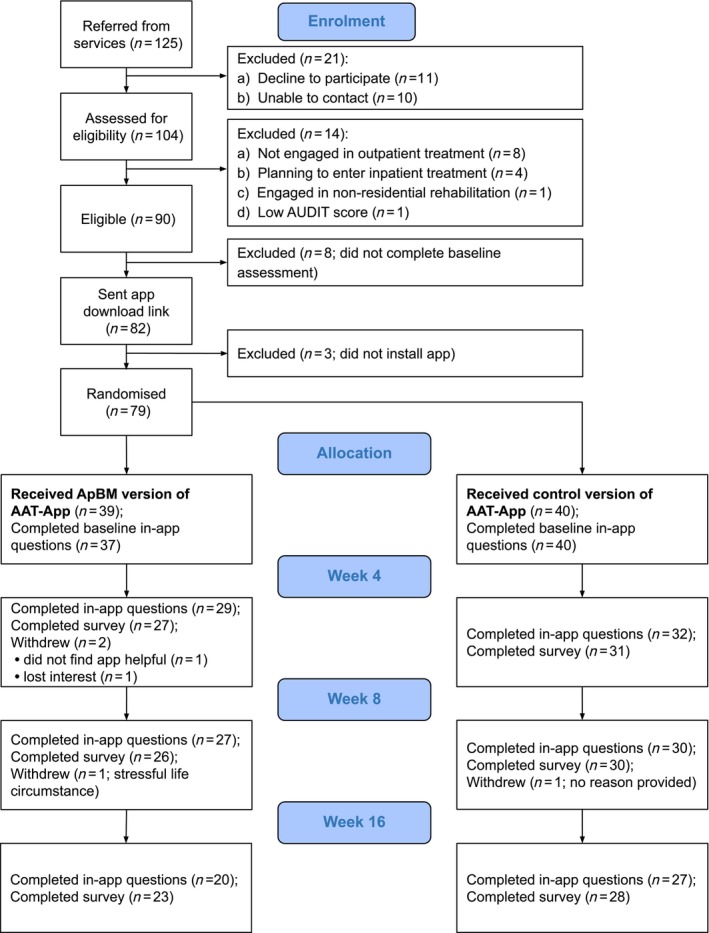
CONSORT flow diagram.

Figure 1 shows numbers of clients referred, screened, randomised and completing each follow‐up. Table 1 presents the sample's baseline demographic characteristics. Table 2 presents baseline clinical characteristics. Participants were mostly White, majority male, with a mean age of 46.6 years (range = 26.6–75.0). Participants in the ApBM condition completed a mean of 8.1 ApBM sessions (range = 1–36) during the 4‐week intervention period. The follow‐up rate for the primary outcome was 77% (missing = 18). At later follow‐ups, alcohol use self‐report was completed by 72% at week 8 (missing = 22), and 59% at week 16 (missing = 32). Two participants did not report alcohol consumption at baseline. For additional outcomes assessed in Qualtrics surveys, follow‐up rates were 73% at week 4, 71% at week 8 and 65% for week 16. These rates did not differ significantly between groups [all χ^2^(1) < 2.16, all *P* > 0.14].

**TABLE 1:** Demographic characteristics of sample at baseline.

Characteristic	Total, *n* = 79	Control, *n* = 40	ApBM, *n* = 39
Age (y)	46.6 (10.3)	44.5 (10.8)	48.8 (9.5)
Gender			
Female	34 (43)	16 (40)	18 (46)
Male	45 (57)	24 (60)	21 (54)
Non‐binary	0 (0)	0 (0)	0 (0)
Race^a^			
Aboriginal or Torres Strait Islander	0 (0)	0 (0)	0 (0)
African	1 (1)	1 (2.5)	0 (0)
East Asian or Southeast Asian	2 (3)	2 (5)	0 (0)
Latino/Latina	2 (3)	2 (5)	0 (0)
South Asian	1 (1)	1 (2.5)	0 (0)
White/European	70 (89)	32 (80)	38 (97)
Prefer not to say	3 (4)	2 (5)	1 (3)
Highest level of completed education			
Less than year 12	12 (15)	4 (10)	8 (21)
Year 12 and/or apprenticeship, diploma or professional qualification	36 (46)	19 (48)	17 (44)
Bachelor's degree or higher	31 (39)	17 (42)	14 (36)
In paid employment	56 (71)	27 (68)	29 (74)
English as first language	77 (97)	38 (95)	39 (100)
Device			
Android	28 (35)	14 (35)	14 (36)
Apple	51 (65)	26 (65)	25 (64)

*Note*: Statistics presented are mean (standard deviation) for age and are n (%) for all other variables.

Abbreviation: ApBM, approach bias modification.

^a^ Participants were allowed the option to select more than one race if they were multi‐racial, however, all participants selected only one option.

**TABLE 2:** Clinical characteristics of sample at baseline.

Characteristic	Total (*n* = 79)	Control (*n* = 40)	ApBM (*n* = 39)
AUDIT score	26.6 (6.2)	26.3 (5.1)	26.9 (7.2)
SDS score	9.1 (3.4)	9.3 (3.0)	8.8 (3.8)
CEQ‐F score	4.3 (2.4)	4.2 (2.4)	4.5 (2.1)
Missing (*n*)	4	2	2
Past‐week standard drinks	36.4 (42.0)	32.4 (34.2)	40.6 (49.1)
Missing (*n*)	2	0	2
Past‐week drinking days	3.3 (2.7)	2.9 (2.6)	3.8 (2.8)
Missing (*n*)	1	0	1
Past‐week heavy drinking days^a^	2.7 (2.6)	2.5 (2.4)	3.0 (2.9)
Missing (*n*)	2	0	2
Past‐week abstinence	28%	33%	24%
Missing (*n*)	1	0	1
ATOP (psychological)	4.6 (2.0)	4.5 (2.0)	4.7 (1.9)
ATOP (physical)	4.9 (2.4)	4.6 (2.5)	5.3 (2.3)
ATOP (quality of life)	5.3 (2.2)	5.0 (2.4)	5.7 (2.0)
Treatment type^b^			
Counselling	88%	87%	89%
Medication	27%	26%	29%
Case management	14%	10%	18%
Missing (*n*)	2	1	1
Time in treatment at time of app installation			
<3 months	73%	76%	71%
3–12 months	19%	14%	24%
>12 months	8%	11%	5%
Missing (*n*)	4	3	1
Treatment goal			
Reduce alcohol consumption	44%	43%	46%
Cease drinking completely	56%	57%	54%
Missing (*n*)	7	5	2
Receiving treatment for other drug(s) in addition to alcohol	17%	15%	19%
Missing (*n*)	2	0	2

*Note*: Statistics presented are mean (SD) except for past‐week abstinence rates, treatment types, treatment duration and whether a participant was receiving treatment for additional drugs, which are percentages. Rows with no *n* for missing data indicate no missing data for that variable.

Abbreviations: ApBM, approach bias modification; ATOP, Australian Treatment Outcomes Profile; AUDIT, Alcohol Use Disorders Identification Test; CEQ‐F, Craving Experience Questionnaire‐Frequency scale; SDS, Severity of Dependence Scale.

^a^ Heavy drinking days were days on which ≥5 standard drinks (i.e. ≥50 g pure alcohol) were consumed.

^b^ Some participants were receiving more than 1 type of treatment simultaneously, hence, percentages add to more than 100%.

### Primary outcome: Past‐week standard drinks

As shown in Table 3, groups did not significantly differ in past‐week standard drinks at week 4 in either ITT or observed‐data analyses.

**TABLE 3:** Primary outcome: Negative binomial mixed model for past‐week standard drinks at week 4.

Analysis	Mean (SE)		Difference	95% CI of difference	β	95% CI of β	*P*
	ApBM	Control					
ITT (*n* = 77)	75.49 (50.40)	71.34 (46.11)	4.16	−42.37 to 50.69	0.06	−0.57 to 0.68	0.859
Observed data (*n* = 60)	53.48 (41.36)	55.69 (44.43)	−2.21	−45.45 to 41.03	−0.04	−0.83 to 0.75	0.920

*Note*: First row shows ITT analysis based on the assumption that outcome data was missing not at random, and hence used last‐outcome‐carried‐forward imputation (i.e. missing outcomes replaced with baseline values). Second row shows analysis with no imputation of missing outcomes. Both analyses controlled for baseline standard drinks. Means (and SEs), differences and 95% CIs of differences, are expected values from fitted negative binomial model.

Abbreviations: ApBM, approach bias modification; ITT, intention‐to‐treat; SE, standard error.

### Secondary outcomes

**FIGURE 2:**
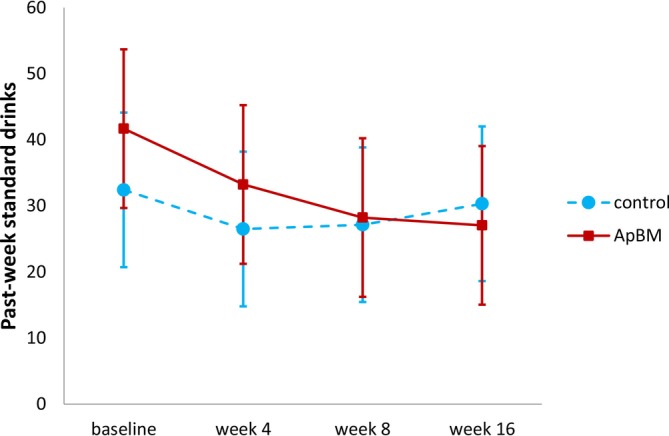
Predicted marginal means of past‐week standard drinks from linear mixed‐effects model testing all timepoints (intention‐to‐treat analysis with last outcome carried forward). Note: a standard drink was defined as a drink containing 10 g of pure ethanol. Error bars represent 95% CI of the mean. ApBM, approach bias modification.

ITT analysis of past‐week standard drinks at later endpoints found that participants in the ApBM group reported significantly greater reductions than control participants at week 16 (relative to baseline), although this difference was non‐significant at week 8 (see Table S1a for effect estimates and Figure 2 for predicted marginal means). Within‐groups pairwise comparisons between time points found that ApBM participants significantly reduced consumption by a mean of 14.63 standard drinks between baseline and 16‐week follow‐up (95% CI = −22.77 to −6.49; *P* < 0.001), while the control group's reduction was not significant (2.10 standard drinks, 95% CI = −9.96 to 5.76; *P* = 0.601). All other secondary outcomes (drinking days; HDDs; abstinence; SDS score; AUDIT score; CEQ score; and ATOP psychological, physical and quality of life rating) showed non‐significant group × time interactions at all time‐points (all *P* > 0.069) (see Supporting information Tables). Significant main effects of time were detected for SDS (at all follow‐ups), AUDIT (at week 16), CEQ‐F (all follow‐ups), ATOP psychological well‐being (at week 8 and 16), ATOP physical wellbeing (week 4 and 16) and ATOP quality of life scores (at all follow‐ups). Hence, these outcomes improved in the control group and ApBM did not significantly modify this effect. Past‐week drinking days, past‐week HDDs and proportion of participants completely abstinent for the past week did not change significantly over time.

**FIGURE 3:**
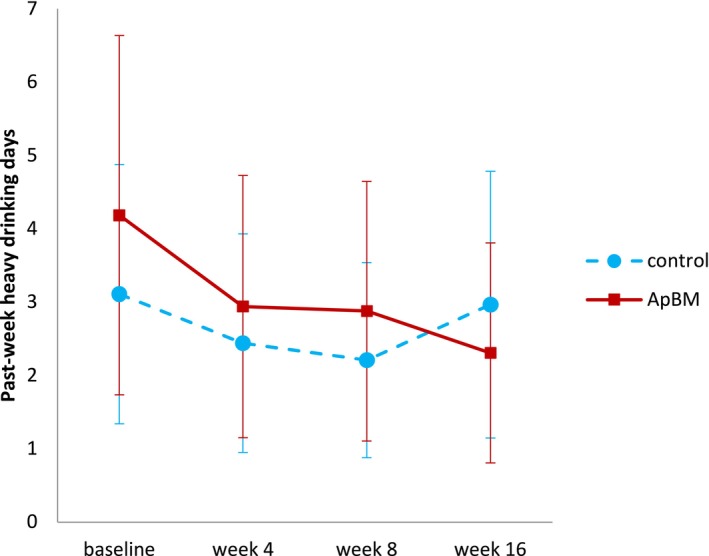
Predicted marginal means of past‐week heavy drinking days across all timepoints (from Poisson model using no imputation of missing outcomes). Note: a heavy drinking day was defined as a day where ≥5 standard drinks (i.e. ≥50 g of pure ethanol) were consumed. Error bars represent 95% CI of the mean. ApBM, approach bias modification.

Complete‐case analyses of secondary outcomes replicated the significant group × time interaction at week 16 for past‐week standard drinks (see Table S1b), and the non‐significance of all other group × time interactions, with the exception of HDDs at week 16 (see Table S3b). Figure 3 suggests this outcome reduced more in the ApBM group than in controls. Pairwise comparisons derived from complete‐case analysis indicated that, between baseline and 16‐week follow‐up, participants in the intervention group significantly reduced HDDs by 0.60 (95% CI = −0.99 to −0.20; *P* = 0.003), while the control group's reduction (0.05) was non‐significant (95% CI = −0.41 to 0.32; *P* = 0.800).

### Subjective ratings of app

Table 4 shows mean scores for uMARS subscales and single‐item subjective app ratings. None of these measures significantly differed between groups. Mean uMARS subscale scores were consistent with positive attitudes toward the app's functionality and aesthetics and neutral attitudes regarding its subjective quality. Mean ratings of the app's perceived effects suggested that, on average, participants felt the training neither substantially reduced nor increased their alcohol consumption or craving. Nine of the 23 participants (39%) in the ApBM group who completed the 16‐week follow‐up, and 16 of the 28 controls (57%) guessed they received the ‘active training’ condition [χ^2^(1) = 1.64, *P* = 0.20].

**TABLE 4:** Means and SDs for scores on the User Version of the Mobile Application Rating Scale subscales and subjective app ratings.

Characteristic	Total, *n* = 58	Control, *n* = 31	ApBM, *n* = 27	Difference	95% CI of difference	*P*
uMARS functionality	4.5 (0.5)	4.5 (0.5)	4.5 (0.6)	0.01	−0.26 to 0.29	0.92
uMARS aesthetics	4.0 (0.7)	4.0 (0.8)	4.1 (0.6)	−0.05	−0.42 to 0.32	0.78
uMARS subjective quality	3.0 (0.9)	2.9 (0.9)	3.1 (0.9)	−0.17	−0.62 to 0.29	0.47
Reduced alcohol consumption	2.8 (0.8)	2.7 (0.8)	2.9 (0.8)	−0.25	−0.68 to 0.18	0.25
Increased alcohol consumption	2.0 (0.9)	2.1 (0.8)	1.9 (1.1)	0.24	−0.26 to 0.74	0.34
Reduced alcohol cravings	2.7 (0.9)	2.6 (0.9)	2.8 (0.8)	−0.20	−0.66 to 0.27	0.40
Increased alcohol cravings	2.2 (1.0)	2.2 (0.9)	2.1 (1.1)	0.08	−0.46 to 0.63	0.76

*Note*: Descriptive statistics presented are mean (SD). *P‐*values are for Welch two sample *t* tests.

Abbreviation: ApBM, approach bias modification; uMARS, User Version of the Mobile Application Rating Scale.

## DISCUSSION

Analysis of our primary outcome did not show any significant effect of providing a personalised ApBM smartphone app, as an adjunctive intervention to people accessing outpatient AUD treatment, on alcohol use after 4 weeks. The majority of secondary outcome analyses also failed to show significant differences between groups in degree of improvement over time. We did, however, find that ApBM participants reduced their alcohol consumption significantly more than controls by the 16‐week follow‐up. Past‐week standard drinks declined significantly in the ApBM group (relative to baseline), but did not change significantly in controls, by this final follow‐up. In addition, complete‐case analysis indicated a similar effect for HDDs, but this was not significant in ITT analysis. This suggests that ApBM possibly had a delayed effect, facilitating reductions in alcohol use over the months following the intervention. Delayed effects are not unprecedented in the broader cognitive bias modification literature. A study of attention bias modification (which is closely related to ApBM) for depression found that training participants to shift attention toward positive facial expressions had no significant effect on depression at the end of the 2‐week intervention, but significantly reduced depression symptoms at a follow‐up 1 month later [31].

There were significant main effects of time for AUDIT and SDS scores, psychological and physical wellbeing, quality of life and craving. In all cases, these outcomes improved over time. However, with the group × time interactions being non‐significant for these outcomes, there was no evidence that the ApBM app enhanced these improvements. Hence, these effects may reflect benefits that both groups received from outpatient treatment and/or non‐specific effects (e.g. regression to the mean, expectancy effects or motivation to make positive changes).

Participants' mean ratings of the app on the uMARS subscales, and the fact that participants in the ApBM condition completed an average of 8.1 ApBM sessions, equivalent to the number (8) that they were prompted to complete by app notifications, support the acceptability of personalised smartphone ApBM in AUD outpatients. Nevertheless, mean ratings of the app's perceived efficacy were relatively low. The lack of significant differences between groups in ratings of the app's quality, perceived efficacy or in the proportion who guessed they were in the ApBM condition, also suggests that a minimal sham‐training control condition successfully achieved blinding and was reasonably acceptable to AUD treatment clients.

Our study was the first (to our knowledge) to test smartphone app‐delivered ApBM in an AUD treatment population, unlike previous smartphone ApBM app studies that used community samples of people drinking heavily [12, 13, 14, 15, 22]. Moreover, to our knowledge, ours is only the second trial to test ApBM in AUD outpatients using any method of ApBM delivery. Our non‐significant primary outcome finding resembles Laurens *et al*.’s [11] finding that, relative to sham‐training controls, keyboard‐based ApBM did not significantly reduce AUD outpatients' alcohol consumption. However, Laurens *et al*. [11] also found no significant effects on alcohol consumption at 3‐month and 6‐month follow‐ups, whereas we found that ApBM reduced alcohol consumption at the 16‐week follow‐up. Laurens *et al*.’s [11] trial differed from ours by delivering ApBM on a computer using non‐personalised stimuli, with participants responding to stimuli by pressing keyboard keys to cause images to shrink or expand. In our study, participants used a smartphone app that allowed users (in the ApBM condition) to personalise their training images, and they responded by swiping images away from or toward, themselves. Embodied approach/avoidance movements that resemble gestures one might use to ‘flick away’ an undesired object, or draw a desired object toward oneself, may more effectively change valuation of stimuli [32]. Laurens *et al*. [11] also used a rigid protocol of eight sessions of training in both groups, while our app allowed participants in the ApBM condition to complete sessions as frequently as they wanted (although it prompted them to complete 8 sessions) and used a more minimal, restricted control condition.

Our reliance on participants' self‐reported estimates of alcohol use may have affected the precision of our data. However, our app's past‐week drinking assessment closely resembled a computerised 7‐day timeline follow‐back assessment used in previous research, which showed 87% concordance with an objective transdermal measure of drinking days [33]. Nevertheless, weekly self‐reporting of alcohol use during the intervention may have confounded this outcome, because minimal assessment of drinking quantity and frequency may cause reductions in use due to ‘assessment reactivity’ [34].

Our small sample size meant our analyses were almost certainly underpowered (with power analysis suggesting only 14% power to detect a modest effect on the primary outcome), limiting the precision of our effect estimates and our ability to detect effects. The move to deliver most outpatient treatment remotely via telehealth during the coronavirus disease (COVID‐19) pandemic reduced the proportion of outpatients visiting treatment sites in‐person, limiting opportunities to inform patients about the research. Moreover, clinicians at some sites informed the research team that many patients were uninterested in participating in research. Although we did not assess clinicians' efforts to inform clients about the research, it is possible that clinicians' ability and willingness to refer clients was limited by lack of time, forgetting to do so, or, as suggested by one recent trial [8], lack of belief in ApBM's efficacy. This suggests that if smartphone ApBM were to be implemented as part of outpatient AUD treatment, implementation research involving clinician and client co‐design would be necessary to understand and overcome patient and clinician barriers to uptake.

The heterogeneity of our sample's clinical and treatment characteristics can be considered both a strength and a weakness of this trial. The substantial variation in participants' baseline alcohol use (e.g. 28% reporting past‐week abstinence at baseline, while others were drinking heavily) and the stage and type of treatment means that our findings cannot clarify important questions relevant to ApBM's implementation among AUD outpatients. Determining whether ApBM's effects depend on baseline alcohol use patterns, stage/type of treatment, *etc*., would require exploration in larger samples. We also did not have access to participants' outpatient treatment records, and therefore, we cannot analyse whether treatment quantity (e.g. number of appointments) was well‐balanced between groups or influenced the effects of the app. However, by imposing relatively minimal eligibility criteria, we recruited a sample with diverse clinical and treatment characteristics, broadening the generalisability of our results.

In conclusion, we did not find evidence that personalised, smartphone‐delivered ApBM reduced alcohol use in AUD outpatients during the 4‐week intervention period. Analysis of secondary outcomes suggested a possible delayed beneficial effect of ApBM at the 16‐week follow‐up, although this should be interpreted cautiously given that this was indicated by just one of several secondary outcomes, the rest showing non‐significant effects. If the positive secondary outcome effect could be replicated in a better‐powered trial, an ApBM app could be easily added to outpatient treatment to enhance treatment outcomes, given the simplicity and brevity of the intervention (4‐minute training sessions). Future research should also aim to clarify moderators of efficacy, such as the optimal stage of outpatient treatment at which to introduce an ApBM app to clients.

## AUTHOR CONTRIBUTIONS

**Joshua B. B. Garfield:** Conceptualization (supporting); data curation (supporting); formal analysis (supporting); funding acquisition (supporting); investigation (equal); methodology (equal); project administration (lead); supervision (lead); writing—original draft (equal); writing—review and editing (lead). **Bosco Rowland:** Formal analysis (lead); writing—review and editing (supporting). **Samuel K. Liu:** Data curation (lead); formal analysis (supporting); project administration (supporting); writing—original draft (equal). **Hugh Piercy:** Conceptualization (supporting); investigation (supporting); methodology (equal); project administration (supporting); writing—review and editing (supporting). **Yvonne Bonomo:** Funding acquisition (supporting); project administration (supporting); resources (supporting); writing—review and editing (supporting). **Danielle Whelan:** Data curation (supporting); investigation (supporting); project administration (supporting); writing—review and editing (supporting). **Victoria Manning:** Conceptualization (lead); funding acquisition (lead); investigation (equal); methodology (equal); project administration (supporting); supervision (supporting); writing—review and editing (supporting).

## DECLARATION OF INTERESTS

All authors declare that they have no competing interests relevant to this manuscript.

## CLINICAL TRIAL REGISTRATION

Trial registration number: NCT05120856. Date of registration: 15 November 2021. Link to registry location: https://clinicaltrials.gov/ct2/show/NCT05120856

## Supporting information


**Table S1a**. Results for linear mixed‐effects model of past‐week standard drinks including all follow‐ups, using intention‐to‐treat analysis
**Table S1b**. Results for linear mixed‐effects model of past‐week standard drinks including all follow‐ups, using only observed data
**Table S2a**. Poisson effects model results for past‐week drinking days, using intention‐to‐treat analysis
**Table S2b**. Poisson effects model results for past‐week drinking days, using only observed data
**Table S3a**. Poisson effects model results for past‐week heavy drinking days, using intention‐to‐treat analysis
**Table S3b**. Poisson effects model results for past‐week heavy drinking days, using only observed data
**Table S4a**. Mixed‐effects logistic regression for past‐week abstinence, using intention‐to‐treat analysis
**Table S4b**. Mixed‐effects logistic regression for past‐week abstinence, using only observed data
**Table S5a**. Linear mixed‐effects model results for the Severity of Dependence Scale scores, using intention‐to‐treat analysis
**Table S5b**. Linear mixed‐effects model results for the Severity of Dependence Scale scores, using only observed data
**Table S6a**. Linear mixed‐effects model results for Alcohol Use Disorder Identification Test scores, using intention‐to‐treat analysis
**Table S6b**. Linear mixed‐effects model results for Alcohol Use Disorder Identification Test scores, using only observed data
**Table S7a**. Linear mixed‐effects model results for Craving Experience Questionnaire frequency scale total scores, using intention‐to‐treat analysis
**Table S7b**. Linear mixed‐effects model results for Craving Experience Questionnaire frequency scale total scores, using only observed data
**Table S8a**. Linear mixed‐effects model results for the Australian Treatment Outcomes Profile psychological wellbeing item, using intention‐to‐treat analysis
**Table S8b**. Linear mixed‐effects model results for the Australian Treatment Outcomes Profile psychological wellbeing item, using only observed data
**Table S9a**. Linear mixed‐effects model results for the Australian Treatment Outcomes Profile physical wellbeing item, using intention‐to‐treat analysis
**Table S9b**. Linear mixed‐effects model results for the Australian Treatment Outcomes Profile physical wellbeing item, using only observed data
**Table S10a**. Linear mixed‐effects model results for the Australian Treatment Outcomes Profile quality of life item, using intention‐to‐treat analysis
**Table S10b**. Linear mixed‐effects model results for the Australian Treatment Outcomes Profile quality of life item, using only observed data

## Data Availability

Researchers interested in accessing deidentified data may contact Victoria Manning at victoria.manning@monash.edu. Granting access to deidentified data to other researchers will require additional approval by the St Vincent's Hospital Melbourne HREC and possibly other HRECs/institutional review boards. Seeking these approvals (including any associated costs) will be the responsibility of the researchers seeking access to the dataset.

## Footnotes

* Participants with multiple drugs of concern were eligible if alcohol was one of the drugs of concern for the current episode of treatment.
