# Supplementary material for: Efficacy of a personalised alcohol approach bias modification smartphone app in people accessing outpatient alcohol use disorder treatment: A randomised controlled trial
Source: Addiction. 2025 Sep 4;121(1):82–93. doi: 10.1111/add.70184 (PMC12710684; doi:10.1111/add.70184)
Supplement: Supplementary file 1 — Table S1a. Results for linear mixed‐effects model of past‐week standard drinks including all follow‐ups, using intention‐to‐treat analysis Table S1b. Results for linear mixed‐effects model of past‐week standard drinks including all follow‐ups, using only observed data Table S2a. Poisson effects model results for past‐week drinking days, using intention‐to‐treat analysis Table S2b. Poisson effects model results for past‐week drinking days, using only observed data Table S3a. Poisson effects model results for past‐week heavy drinking days, using intention‐to‐treat analysis Table S3b. Poisson effects model results for past‐week heavy drinking days, using only observed data Table S4a. Mixed‐effects logistic regression for past‐week abstinence, using intention‐to‐treat analysis Table S4b. Mixed‐effects logistic regression for past‐week abstinence, using only observed data Table S5a. Linear mixed‐effects model results for the Severity of Dependence Scale scores, using intention‐to‐treat analysis Table S5b. Linear mixed‐effects model results for the Severity of Dependence Scale scores, using only observed data Table S6a. Linear mixed‐effects model results for Alcohol Use Disorder Identification Test scores, using intention‐to‐treat analysis Table S6b. Linear mixed‐effects model results for Alcohol Use Disorder Identification Test scores, using only observed data Table S7a. Linear mixed‐effects model results for Craving Experience Questionnaire frequency scale total scores, using intention‐to‐treat analysis Table S7b. Linear mixed‐effects model results for Craving Experience Questionnaire frequency scale total scores, using only observed data Table S8a. Linear mixed‐effects model results for the Australian Treatment Outcomes Profile psychological wellbeing item, using intention‐to‐treat analysis Table S8b. Linear mixed‐effects model results for the Australian Treatment Outcomes Profile psychological wellbeing item, using only observed data Table S9a. Linear mixed‐effects mo [file ADD-121-82-s001.docx]

**Supplementary Methods**

**Intervention**

*ApBM condition*: When they first installed the app, participants in the ApBM condition viewed a short video explaining how ApBM is purported to work. They were then prompted to select 6 alcohol-related images that represented the drinks they most frequently consumed. They could choose from 67 images provided in the app depicting alcoholic beverages common in Australia or could use images from their phone’s photo library or by using their phone camera. Next, participants selected 6 positive images representing healthy activities and positive goals. They could choose from 68 images provided in the app (e.g., depicting family activities, financial success, employment, exercise, etc.) or use photos stored on their phone. If participants used photos from their own phone as positive imagery, the app reminded them to avoid using photos that contained alcohol-related imagery.

After selecting their 12 images, participants were presented with ApBM task instructions. Each trial involved an image being presented within a white frame in either landscape or portrait orientation. Participants were instructed to swipe images framed in landscape orientation towards themselves (i.e., swiping their finger downwards on their phone screen), while swiping images framed in portrait orientation away from themselves (i.e., upwards). Swiping downwards caused the image to expand, while swiping upwards caused it to shrink and disappear, to enhance the perception of “approach” and “avoidance”.

Participants completed 10 practice trials before proceeding to the first session of ApBM. Each session consisted of 156 trials, comprising 13 presentations of each of the 12 selected images, in a quasi-random order. For alcohol images, 12 of the 13 presentations were framed in portrait orientation, and one was framed in landscape orientation. For positive pictures, 12 presentations were framed in landscape orientation, while one was framed in portrait orientation. Thus, participants were supposed to “push away” 92.3% of alcohol images and “pull” 92.3% of positive images towards themselves. If participants made the incorrect response, a red “X” was displayed to inform them of their error, but the trial was not repeated.

To increase engagement and encourage participants to respond both quickly and accurately, the task was gamified with a scoring system. If they responded correctly, they earned points for that trial based on how quickly they responded (40 points for responses faster than 500 ms, 30 points for responses with 501-1000 ms latency, 20 points for 1001-1500 ms, or 10 points for responses slower than 1500 ms). If they responded incorrectly, they lost 100 points, regardless of speed of response. Participants’ score was displayed on the screen as they performed the task. Upon completion of the task, the final score was displayed. On the second, and subsequent, sessions, participants’ previous session score, and the score of their highest-scoring session, were displayed before commencing the task, to encourage them to try to score higher. At the end of these sessions, their previous best score was displayed alongside the score for the session they just completed, so they could see whether or not they had exceeded their previous best score.

App notifications reminded participants to complete 2 training sessions each week during the 4-week intervention period. They could complete more sessions if they wished, and could continue accessing the training task after the end of the 4-week intervention. However, notifications did not continue beyond the 4^th^ week.

*Control condition*: In the control condition, when participants first installed the app, text information told participants that the app was “designed to target their alcohol use and awareness”. They were then presented with task instructions, which were identical to those in ApBM (i.e., swipe images in landscape orientation towards themselves; swipe images in portrait orientation away from themselves), and they then completed 10 practice trials before commencing their first session of sham training. Sham training involved presentation of 20 images of alcohol and 20 neutral images (e.g., stationary, kitchenware), and images were not personalised (i.e., the same 40 images were used for all participants in the sham-training control group). Each image was presented twice per session, once surrounded by a frame in landscape orientation, and once in a portrait-oriented frame (for a total of 80 image presentations), in a quasi-random order. Hence, participants were supposed to swipe 50% of alcohol images and 50% of neutral images away; and swipe 50% of each category towards themselves. Following completion of their first session, participants were instructed that they would be prompted to complete additional “brain-training” tasks at the end of each of the following 4 weeks. They could not complete additional sessions, and could only complete each weekly session after they became “unlocked” at 7-day intervals.

This control condition primarily aimed to blind participants to condition allocation by providing a weekly task that could be presented as “training”, while minimising the likelihood of the sham training having clinically relevant effects, other than non-specific placebo/expectancy effects, itself (e.g., directly affecting alcohol approach). Previous ApBM RCTs in AUD patients have generally either included control groups that received no training task (i.e., treatment as usual only) or that received sham training that was matched to ApBM training in terms of number of sessions and number of trials per session. Studies that included both no-training and sham-training conditions found that these controls groups did not have significantly different alcohol use outcomes (1, 2). However, it has been argued that sham-training conditions, despite being intended not to modify approach bias (because they involve approaching and avoiding alcohol stimuli an equal number of times), could theoretically reduce approach bias in people with AUD nevertheless. If someone has a strong tendency to approach alcohol stimuli *every* time they are presented (or even most of the time), training them to avoid these stimuli 50% of the time may still weaken their approach bias (3, 4). Hence, while we decided to include a typical sham-training task in the control condition in the hopes that this would help blind participants to their condition allocation, we limited the number of times they could complete it (to once per week) and the number of trials per session (80 in the sham-training task, rather than 156 as in the ApBM task) to minimise the likelihood of training effects that could confound our ability to detect ApBM’s effects.

**Supplementary Tables**

**Table S1a** *Results for linear mixed-effects model of past-week standard drinks including all follow-ups, using intention-to-treat analysis*

| *Predictors* | *Estimates* | *SE* | *z* | *95% CI* | *p* |
| --- | --- | --- | --- | --- | --- |
| (Intercept) | 32.42 | 5.97 | 5.43 | 20.73, 44.12 | **<.001** |
| Time |  |  |  |  |  |
| Week 4 | -5.92 | 4.01 | -1.48 | -13.79, 1.94 | .140 |
| Week 8 | -5.28 | 4.01 | -1.31 | -13.14, 2.59 | .189 |
| Week 16 | -2.10 | 4.01 | -0.52 | -9.96, 5.76 | .601 |
| Condition | 9.26 | 8.57 | 1.08 | -7.53, 26.05 | .280 |
| Timepoint x Condition |  |  |  |  |  |
| Week 4 x Condition | -2.53 | 5.77 | -0.44 | -13.84, 8.79 | .662 |
| Week 8 x Condition | -8.18 | 5.77 | -1.42 | -19.49, 3.14 | .157 |
| Week 16 x Condition | -12.53 | 5.77 | -2.17 | -23.85, -1.22 | **.030** |
| Random Effects | | | | | |
| Intercept variance | 1102.49 | | | | |
| Residual variance | 322.00 | | | | |
| Participants | 78 | | | | |
| Observations | 311 | | | | |

*Note*. Values highlighted in bold indicate p-values that are statistically significant at an alpha level of .05. CI = confidence interval; SE = standard error.

**Table S1b** *Results for linear mixed-effects model of past-week standard drinks including all follow-ups, using only observed data*

| *Predictors* | *Estimates* | *SE* | *z* | *95% CI* | *p* |
| --- | --- | --- | --- | --- | --- |
| (Intercept) | 32.42 | 5.71 | 5.68 | 21.24, 43.61 | **<.001** |
| Time |  |  |  |  |  |
| Week 4 | -7.95 | 5.03 | -1.58 | -17.80, 1.90 | .114 |
| Week 8 | -5.72 | 5.14 | -1.11 | -15.79, 4.35 | .265 |
| Week 16 | -4.50 | 5.34 | -0.84 | -14.96, 5.96 | .399 |
| Condition | 9.32 | 8.20 | 1.14 | -6.75, 25.39 | .256 |
| Timepoint x Condition |  |  |  |  |  |
| Week 4 x Condition | -4.29 | 7.33 | -0.59 | -18.65, 10.07 | .558 |
| Week 8 x Condition | -12.67 | 7.51 | -1.69 | -27.39, 2.06 | .092 |
| Week 16 x Condition | -16.24 | 8.11 | -2.00 | -32.13, -0.35 | **.045** |
| Random Effects | | | | | |
| Intercept variance | 876.88 | | | | |
| Residual variance | 425.14 | | | | |
| Participants | 78 | | | | |
| Observations | 242 | | | | |

*Note*. Values highlighted in bold indicate p-values that are statistically significant at an alpha level of .05. CI = confidence interval; SE = standard error.

**Table S2a** *Poisson effects model results for past-week drinking days, using intention-to-treat analysis*

| *Predictors* | *Estimates* | *SE* | *z* | *95% CI* | *p* |
| --- | --- | --- | --- | --- | --- |
| (Intercept) | 0.45 | 0.24 | 1.90 | -0.01, 0.92 | .057 |
| Time |  |  |  |  |  |
| Week 4 | -0.03 | 0.13 | -0.20 | -0.29, 0.23 | .843 |
| Week 8 | -0.02 | 0.13 | -0.13 | -0.28, 0.24 | .895 |
| Week 16 | 0.07 | 0.13 | 0.58 | -0.18, 0.33 | .562 |
| Condition | 0.42 | 0.33 | 1.28 | -0.22, 1.06 | .200 |
| Timepoint x Condition |  |  |  |  |  |
| Week 4 x Condition | -0.10 | 0.18 | -0.55 | -0.45, 0.25 | .583 |
| Week 8 x Condition | -0.11 | 0.18 | -0.60 | -0.46, 0.24 | .549 |
| Week 16 x Condition | -0.20 | 0.18 | -1.13 | -0.55, 0.15 | .260 |
| Random Effects | | | | | |
| Intercept variance | 1.67 | | | | |
| Participants | 78 | | | | |
| Observations | 312 | | | | |

*Note*. CI = confidence interval; SE = standard error.

**Table S2b** *Poisson effects model results for past-week drinking days, using only observed data*

| *Predictors* | *Estimates* | *SE* | *z* | *95% CI* | *p* |
| --- | --- | --- | --- | --- | --- |
| (Intercept) | 0.55 | 0.22 | 2.50 | 0.12, 0.98 | **.013** |
| Time |  |  |  |  |  |
| Week 4 | -0.01 | 0.15 | -0.07 | -0.31, 0.29 | .945 |
| Week 8 | -0.01 | 0.15 | -0.07 | -0.29, 0.27 | .946 |
| Week 16 | 0.07 | 0.16 | 0.46 | -0.24, 0.38 | .646 |
| Condition | 0.38 | 0.30 | 1.27 | -0.21, 0.97 | .205 |
| Timepoint x Condition |  |  |  |  |  |
| Week 4 x Condition | -0.18 | 0.21 | -0.89 | -0.59, 0.22 | .372 |
| Week 8 x Condition | -0.19 | 0.20 | -0.92 | -0.59, 0.21 | .358 |
| Week 16 x Condition | -0.27 | 0.23 | -1.21 | -0.72, 0.17 | .225 |
| Random Effects | | | | | |
| Intercept variance | 1.33 | | | | |
| Participants | 78 | | | | |
| Observations | 243 | | | | |

*Note*. Values highlighted in bold indicate p-values that are statistically significant at an alpha level of .05. CI = confidence interval; SE = standard error.

**Table S3a** *Poisson effects model results for past-week heavy drinking days, using intention-to-treat analysis*

| *Predictors* | *Estimates* | *SE* | *z* | *95% CI* | *p* |
| --- | --- | --- | --- | --- | --- |
| (Intercept) | 0.12 | 0.27 | 0.46 | -0.41, 0.65 | .647 |
| Time |  |  |  |  |  |
| Week 4 | -0.18 | 0.15 | -1.19 | -0.47, 0.12 | .234 |
| Week 8 | -0.27 | 0.15 | -1.74 | -0.57, 0.03 | .081 |
| Week 16 | -0.04 | 0.14 | -0.29 | -0.32, 0.24 | .773 |
| Condition | 0.36 | 0.37 | 0.97 | -0.37, 1.08 | .334 |
| Timepoint x Condition |  |  |  |  |  |
| Week 4 x Condition | -0.04 | 0.20 | -0.22 | -0.45, 0.36 | .829 |
| Week 8 x Condition | 0.03 | 0.21 | 0.17 | -0.37, 0.44 | .869 |
| Week 16 x Condition | -0.30 | 0.20 | -1.48 | -0.70, 0.10 | .139 |
| Random Effects | | | | | |
| Intercept variance | 2.12 | | | | |
| Participants | 78 | | | | |
| Observations | 311 | | | | |

*Note*. CI = confidence interval; SE = standard error.

**Table S3b** *Poisson effects model results for past-week heavy drinking days, using only observed data*

| *Predictors* | *Estimates* | *SE* | *z* | *95% CI* | *p* |
| --- | --- | --- | --- | --- | --- |
| (Intercept) | 0.23 | 0.26 | 0.90 | -0.27, 0.73 | .366 |
| Time |  |  |  |  |  |
| Week 4 | -0.24 | 0.18 | -1.32 | -0.60, 0.12 | .188 |
| Week 8 | -0.34 | 0.18 | -1.94 | -0.69, 0.003 | .052 |
| Week 16 | -0.05 | 0.19 | -0.25 | -0.41, 0.32 | .800 |
| Condition | 0.30 | 0.35 | 0.85 | -0.39, 0.98 | .393 |
| Timepoint x Condition |  |  |  |  |  |
| Week 4 x Condition | -0.11 | 0.24 | -0.45 | -0.59, 0.37 | .651 |
| Week 8 x Condition | -0.03 | 0.24 | -0.14 | -0.51, 0.44 | .892 |
| Week 16 x Condition | -0.55 | 0.27 | -2.01 | -1.08, -0.01 | **.045** |
| Random Effects | | | | | |
| Intercept variance | 1.81 | | | | |
| Participants | 78 | | | | |
| Observations | 242 | | | | |

*Note*. Values highlighted in bold indicate p-values that are statistically significant at an alpha level of .05. CI = confidence interval; SE = standard error.

**Table S4a** *Mixed-effects logistic regression for past-week abstinence, using intention-to-treat analysis*

| *Predictors* | *Estimates (OR)* | | *SE* | *z* | *95% CI* | *p* | |
| --- | --- | --- | --- | --- | --- | --- | --- |
| (Intercept) | 0.48 | | 0.16 | -2.16 | 0.25, 0.93 | **.031** | |
| Time |  | |  |  |  |  | |
| Week 4 | 1.25 | | 0.59 | 0.47 | 0.50, 3.13 | .640 | |
| Week 8 | 1.38 | | 0.65 | 0.70 | 0.55, 3.46 | .487 | |
| Week 16 | 1.12 | | 0.53 | 0.24 | 0.44, 2.83 | .813 | |
| Condition | 0.64 | | 0.33 | -0.86 | 0.24, 1.75 | .389 | |
| Timepoint x Condition |  | |  |  |  |  | |
| Week 4 x Condition | 0.80 | | 0.58 | -0.31 | 0.20, 3.27 | .759 | |
| Week 8 x Condition | 1.07 | | 0.75 | 0.10 | 0.27, 4.22 | .918 | |
| Week 16 x Condition | 1.17 | | 0.83 | 0.23 | 0.29, 4.69 | .821 | |
| Participants | | 78 | | | | |  |
| Observations | | 312 | | | | |  |

*Note*. Values highlighted in bold indicate p-values that are statistically significant at an alpha level of .05. CI = confidence interval; OR = odds ratio; SE = standard error. As mixed effects model would not converge, this model uses logistic regression with robust standard errors.

**Table S4b** *Mixed-effects logistic regression for past-week abstinence, using only observed data*

| *Predictors* | *Estimates (OR)* | *SE* | *z* | *95% CI* | *p* |
| --- | --- | --- | --- | --- | --- |
| (Intercept) | .09 | .09 | -2.46 | 0.01, 0.61 | **.014** |
| Time |  |  |  |  |  |
| Week 4 | 1.95 | 1.69 | 0.77 | 0.35, 10.70 | .444 |
| Week 8 | 3.05 | 2.71 | 1.26 | 0.54, 17.34 | .208 |
| Week 16 | 1.21 | 1.11 | 0.21 | 0.20, 7.23 | .831 |
| Condition | 0.32 | 0.40 | -0.92 | .03, 3.59 | .357 |
| Timepoint x Condition |  |  |  |  |  |
| Week 4 x Condition | 0.68 | 0.87 | -0.30 | 0.06, 8.26 | .765 |
| Week 8 x Condition | 1.59 | 2.04 | 0.36 | 0.13, 19.49 | .715 |
| Week 16 x Condition | 3.12 | 4.22 | 0.84 | 0.22, 44.36 | .401 |
| Random Effects | | | | | |
| Residual variance | 22.21 | | | | |
| Participants | 78 | | | | |
| Observations | 243 | | | | |

*Note*. Values highlighted in bold indicate p-values that are statistically significant at an alpha level of .05. CI = confidence interval; OR = odds ratio; SE = standard error.

**Table S5a** *Linear mixed-effects model results for the Severity of Dependence Scale scores, using intention-to-treat analysis*

| *Predictors* | *Estimates* | *SE* | *z* | *95% CI* | *p* |
| --- | --- | --- | --- | --- | --- |
| (Intercept) | 9.32 | 0.62 | 15.07 | 8.11, 10.54 | **<.001** |
| Time |  |  |  |  |  |
| Week 4 | -2.12 | 0.55 | -3.85 | -3.21, -1.04 | **<.001** |
| Week 8 | -2.28 | 0.55 | -4.12 | -3.36, -1.19 | **<.001** |
| Week 16 | -3.40 | 0.55 | -6.16 | -4.48, -2.32 | **<.001** |
| Condition | -0.53 | 0.88 | -0.60 | -2.26, 1.20 | .547 |
| Timepoint x Condition |  |  |  |  |  |
| Week 4 x Condition | 0.23 | 0.79 | 0.29 | -1.31, 1.77 | .772 |
| Week 8 x Condition | 0.17 | 0.79 | 0.22 | -1.37, 1.71 | .826 |
| Week 16 x Condition | 1.43 | 0.79 | 1.81 | -0.11, 2.97 | .070 |
| Random Effects | | | | | |
| Intercept variance | 9.21 | | | | |
| Residual variance | 6.10 | | | | |
| Participants | 79 | | | | |
| Observations | 316 | | | | |

*Note*. Values highlighted in bold indicate p-values that are statistically significant at an alpha level of .05. CI = confidence interval; SE = standard error.

**Table S5b** *Linear mixed-effects model results for the Severity of Dependence Scale scores, using only observed data*

| *Predictors* | *Estimates* | *SE* | *z* | *95% CI* | *p* |
| --- | --- | --- | --- | --- | --- |
| (Intercept) | 9.32 | 0.60 | 15.58 | 8.15, 10.50 | **<.001** |
| Time |  |  |  |  |  |
| Week 4 | -2.46 | 0.68 | -3.61 | -3.80, -1.13 | **<.001** |
| Week 8 | -2.57 | 0.69 | -3.73 | -3.93, -1.22 | **<.001** |
| Week 16 | -3.96 | 0.70 | -5.68 | -5.33, -2.59 | **<.001** |
| Condition | -0.53 | 0.85 | -0.62 | -2.20, 1.14 | .534 |
| Timepoint x Condition |  |  |  |  |  |
| Week 4 x Condition | -0.23 | 0.99 | -0.23 | -2.17, 1.72 | .820 |
| Week 8 x Condition | -0.13 | 1.01 | -0.13 | -2.11, 1.84 | .896 |
| Week 16 x Condition | 1.42 | 1.04 | 1.37 | -0.62, 3.45 | .172 |
| Random Effects | | | | | |
| Intercept variance | 6.55 | | | | |
| Residual variance | 7.78 | | | | |
| Participants | 79 | | | | |
| Observations | 245 | | | | |

*Note*. Values highlighted in bold indicate p-values that are statistically significant at an alpha level of .05. CI = confidence interval; SE = standard error.

**Table S6a** *Linear mixed-effects model results for Alcohol Use Disorder Identification Test scores, using intention-to-treat analysis*

| *Predictors* | *Estimates* | *SE* | *z* | *95% CI* | *p* |
| --- | --- | --- | --- | --- | --- |
| (Intercept) | 26.25 | 1.38 | 18.99 | 23.54, 28.96 | **<.001** |
| Time | -9.12 | 1.68 | -5.43 | -12.42, -5.83 | **<.001** |
| Condition | 0.65 | 1.97 | 0.33 | -3.21, 4.50 | .742 |
| Timepoint x Condition | 1.18 | 2.39 | 0.49 | -3.51, 5.86 | .623 |
| Random Effects | | | | | |
| Intercept variance | 20.04 | | | | |
| Residual variance | 56.39 | | | | |
| Participants | 79 | | | | |
| Observations | 158 | | | | |

*Note*. Values highlighted in bold indicate p-values that are statistically significant at an alpha level of .05. CI = confidence interval; SE = standard error.

**Table S6b** *Linear mixed-effects model results for Alcohol Use Disorder Identification Test scores, using only observed data*

| *Predictors* | *Estimates* | *SE* | *z* | *95% CI* | *p* |
| --- | --- | --- | --- | --- | --- |
| (Intercept) | 26.25 | 1.28 | 20.52 | 23.74, 28.76 | **<.001** |
| Time | -12.45 | 1.80 | -6.92 | -15.98, -8.93 | **<.001** |
| Condition | 0.65 | 1.82 | 0.36 | -2.92, 4.22 | .722 |
| Timepoint x Condition | -0.42 | 2.66 | -0.16 | -5.62, 4.79 | .876 |
| Random Effects | | | | | |
| Intercept variance | 12.69 | | | | |
| Residual variance | 52.77 | | | | |
| Participants | 79 | | | | |
| Observations | 131 | | | | |

*Note*. Values highlighted in bold indicate p-values that are statistically significant at an alpha level of .05. CI = confidence interval; SE = standard error.

**Table S7a** *Linear mixed-effects model results for Craving Experience Questionnaire frequency scale total scores, using intention-to-treat analysis*

| *Predictors* | *Estimates* | *SE* | *z* | *95% CI* | *p* |
| --- | --- | --- | --- | --- | --- |
| (Intercept) | 4.14 | 0.35 | 11.91 | 3.46, 4.82 | **<.001** |
| Time |  |  |  |  |  |
| Week 4 | -0.71 | 0.28 | -2.55 | -1.26, -0.16 | **.011** |
| Week 8 | -0.57 | 0.28 | -2.05 | -1.12, -0.03 | **.040** |
| Week 16 | -0.94 | 0.28 | -3.36 | -1.48, -0.39 | **.001** |
| Condition | 0.35 | 0.50 | 0.72 | -0.62, 1.33 | .475 |
| Timepoint x Condition |  |  |  |  |  |
| Week 4 x Condition | -0.25 | 0.40 | -0.62 | -1.03, 0.53 | .536 |
| Week 8 x Condition | -0.48 | 0.40 | -1.20 | -1.26, 0.30 | .228 |
| Week 16 x Condition | -0.16 | 0.40 | -0.41 | -0.94, 0.62 | .683 |
| Random Effects | | | | | |
| Intercept variance | 3.21 | | | | |
| Residual variance | 1.50 | | | | |
| Participants | 78 | | | | |
| Observations | 307 | | | | |

*Note*. Values highlighted in bold indicate p-values that are statistically significant at an alpha level of .05. CI = confidence interval; SE = standard error.

**Table S7b** *Linear mixed-effects model results for Craving Experience Questionnaire frequency scale total scores, using only observed data*

| *Predictors* | *Estimates* | *SE* | *z* | *95% CI* | *p* |
| --- | --- | --- | --- | --- | --- |
| (Intercept) | 4.15 | 0.35 | 11.97 | 3.47, 4.83 | **<.001** |
| Time |  |  |  |  |  |
| Week 4 | -0.89 | 0.34 | -2.66 | -1.55, -0.23 | **.008** |
| Week 8 | -0.62 | 0.34 | -1.82 | -1.29, 0.05 | .068 |
| Week 16 | -1.24 | 0.35 | -3.54 | -1.93, -0.55 | **<.001** |
| Condition | 0.35 | 0.50 | 0.71 | -0.62, 1.32 | .480 |
| Timepoint x Condition |  |  |  |  |  |
| Week 4 x Condition | -0.46 | 0.49 | -0.93 | -1.42, 0.51 | .352 |
| Week 8 x Condition | -0.78 | 0.50 | -1.57 | -1.76, 0.19 | .115 |
| Week 16 x Condition | -0.21 | 0.52 | -0.41 | -1.22, 0.80 | .681 |
| Random Effects | | | | | |
| Intercept variance | 2.86 | | | | |
| Residual variance | 1.82 | | | | |
| Participants | 78 | | | | |
| Observations | 240 | | | | |

*Note*. Values highlighted in bold indicate p-values that are statistically significant at an alpha level of .05. CI = confidence interval; SE = standard error.

**Table S8a** *Linear mixed-effects model results for the Australian Treatment Outcomes Profile psychological wellbeing item, using intention-to-treat analysis*

| *Predictors* | *Estimates* | *SE* | *z* | *95% CI* | *p* |
| --- | --- | --- | --- | --- | --- |
| (Intercept) | 4.48 | 0.34 | 13.25 | 3.81, 5.14 | **<.001** |
| Time |  |  |  |  |  |
| Week 4 | 0.58 | 0.32 | 1.81 | -0.05, 1.20 | .070 |
| Week 8 | 0.72 | 0.32 | 2.28 | 0.10, 1.35 | .**022** |
| Week 16 | 0.78 | 0.32 | 2.44 | 0.15, 1.40 | .015 |
| Condition | 0.19 | 0.48 | 0.40 | -0.75, 1.13 | .690 |
| Timepoint x Condition |  |  |  |  |  |
| Week 4 x Condition | -0.32 | 0.45 | -0.70 | -1.20, 0.57 | .481 |
| Week 8 x Condition | -0.37 | 0.45 | -0.81 | -1.25, 0.52 | .418 |
| Week 16 x Condition | -0.80 | 0.45 | -1.77 | -1.69, 0.09 | .077 |
| Random Effects | | | | | |
| Intercept variance | 2.54 | | | | |
| Residual variance | 2.02 | | | | |
| Participants | 79 | | | | |
| Observations | 316 | | | | |

*Note*. Values highlighted in bold indicate p-values that are statistically significant at an alpha level of .05. CI = confidence interval; SE = standard error.

**Table S8b** *Linear mixed-effects model results for the Australian Treatment Outcomes Profile psychological wellbeing item, using only observed data*

| *Predictors* | *Estimates* | *SE* | *z* | *95% CI* | *p* |
| --- | --- | --- | --- | --- | --- |
| (Intercept) | 4.48 | 0.34 | 13.32 | 3.82, 5.13 | **<.001** |
| Time |  |  |  |  |  |
| Week 4 | 0.70 | 0.40 | 1.76 | -0.08, 1.49 | .078 |
| Week 8 | 0.95 | 0.40 | 2.36 | 0.16, 1.75 | **.018** |
| Week 16 | 0.91 | 0.41 | 2.23 | 0.11, 1.71 | **.026** |
| Condition | 0.19 | 0.48 | 0.40 | -0.75, 1.13 | .688 |
| Timepoint x Condition |  |  |  |  |  |
| Week 4 x Condition | -0.17 | 0.58 | -0.28 | -1.31, 0.98 | .777 |
| Week 8 x Condition | -0.31 | 0.59 | -0.52 | -1.46, 0.85 | .600 |
| Week 16 x Condition | -0.88 | 0.61 | -1.46 | -2.07, 0.31 | .145 |
| Random Effects | | | | | |
| Intercept variance | 1.83 | | | | |
| Residual variance | 2.68 | | | | |
| Participants | 79 | | | | |
| Observations | 245 | | | | |

*Note*. Values highlighted in bold indicate p-values that are statistically significant at an alpha level of .05. CI = confidence interval; SE = standard error.

**Table S9a** *Linear mixed-effects model results for the Australian Treatment Outcomes Profile physical wellbeing item, using intention-to-treat analysis*

| *Predictors* | *Estimates* | *SE* | *z* | *95% CI* | *p* |
| --- | --- | --- | --- | --- | --- |
| (Intercept) | 4.58 | 0.36 | 12.75 | 3.87, 5.28 | <**.001** |
| Time |  |  |  |  |  |
| Week 4 | 0.62 | 0.31 | 2.05 | 0.03, 1.22 | **.041** |
| Week 8 | 0.45 | 0.31 | 1.47 | -0.15, 1.05 | .141 |
| Week 16 | 0.70 | 0.31 | 2.29 | 0.10, 1.30 | **.022** |
| Condition | 0.71 | 0.51 | 1.38 | -0.29, 1.71 | .166 |
| Timepoint x Condition |  |  |  |  |  |
| Week 4 x Condition | -0.83 | 0.43 | -1.91 | -1.68, 0.02 | .056 |
| Week 8 x Condition | -0.27 | 0.43 | -0.62 | -1.12, 0.58 | .534 |
| Week 16 x Condition | -0.78 | 0.43 | -1.79 | -1.63, 0.08 | .074 |
| Random Effects | | | | | |
| Intercept variance | 3.28 | | | | |
| Residual variance | 1.87 | | | | |
| Participants | 79 | | | | |
| Observations | 316 | | | | |

*Note*. Values highlighted in bold indicate p-values that are statistically significant at an alpha level of .05. CI = confidence interval; SE = standard error.

**Table S9b** *Linear mixed-effects model results for the Australian Treatment Outcomes Profile physical wellbeing item, using only observed data*

| *Predictors* | *Estimates* | *SE* | *z* | *95% CI* | *p* |
| --- | --- | --- | --- | --- | --- |
| (Intercept) | 4.58 | 0.35 | 12.96 | 3.88, 5.27 | **<.001** |
| Time |  |  |  |  |  |
| Week 4 | 0.90 | 0.38 | 2.37 | 0.15, 1.65 | **.018** |
| Week 8 | 0.46 | 0.39 | 1.18 | -0.30, 1.21 | .236 |
| Week 16 | 0.80 | 0.39 | 2.05 | 0.04, 1.57 | **.040** |
| Condition | 0.71 | 0.50 | 1.41 | -0.28, 1.69 | .159 |
| Timepoint x Condition |  |  |  |  |  |
| Week 4 x Condition | -1.02 | 0.56 | -1.83 | -2.11, 0.07 | .068 |
| Week 8 x Condition | -0.03 | 0.56 | -0.06 | -1.14, 1.07 | .953 |
| Week 16 x Condition | -0.75 | 0.58 | -1.29 | -1.89, 0.39 | .197 |
| Random Effects | | | | | |
| Intercept variance | 2.57 | | | | |
| Residual variance | 2.42 | | | | |
| Participants | 79 | | | | |
| Observations | 245 | | | | |

*Note*. Values highlighted in bold indicate p-values that are statistically significant at an alpha level of .05. CI = confidence interval; SE = standard error.

**Table S10a** *Linear mixed-effects model results for the Australian Treatment Outcomes Profile quality of life item, using intention-to-treat analysis*

| *Predictors* | *Estimates* | *SE* | *z* | *95% CI* | *p* |
| --- | --- | --- | --- | --- | --- |
| (Intercept) | 5.00 | 0.36 | 14.06 | 4.30, 5.70 | **<.001** |
| Time |  |  |  |  |  |
| Week 4 | 0.80 | 0.29 | 2.78 | 0.24, 1.36 | **.005** |
| Week 8 | 0.65 | 0.29 | 2.26 | 0.09, 1.21 | **.024** |
| Week 16 | 0.88 | 0.29 | 3.04 | 0.31, 1.44 | **.002** |
| Condition | 0.69 | 0.51 | 1.37 | -0.30, 1.68 | .171 |
| Timepoint x Condition |  |  |  |  |  |
| Week 4 x Condition | -0.70 | 0.41 | -1.70 | -1.50, 0.10 | .088 |
| Week 8 x Condition | -0.57 | 0.41 | -1.40 | -1.38, 0.23 | .161 |
| Week 16 x Condition | -0.57 | 0.41 | -1.39 | -1.37, 0.23 | .166 |
| Random Effects | | | | | |
| Intercept variance | 3.40 | | | | |
| Residual variance | 1.65 | | | | |
| Participants | 79 | | | | |
| Observations | 316 | | | | |

*Note*. Values highlighted in bold indicate p-values that are statistically significant at an alpha level of .05. CI = confidence interval; SE = standard error.

**Table S10b** *Linear mixed-effects model results for the Australian Treatment Outcomes Profile quality of life item, using only observed data*

| *Predictors* | *Estimates* | *SE* | *z* | *95% CI* | *p* |
| --- | --- | --- | --- | --- | --- |
| (Intercept) | 5.00 | 0.35 | 14.40 | 4.32, 5.68 | **<.001** |
| Time |  |  |  |  |  |
| Week 4 | 0.89 | 0.37 | 2.44 | 0.17, 1.61 | **.015** |
| Week 8 | 0.78 | 0.37 | 2.11 | 0.05, 1.50 | **.035** |
| Week 16 | 1.15 | 0.37 | 3.09 | 0.42, 1.89 | **.002** |
| Condition | 0.69 | 0.49 | 1.40 | -0.28, 1.66 | .161 |
| Timepoint x Condition |  |  |  |  |  |
| Week 4 x Condition | -0.64 | 0.53 | -1.21 | -1.69, 0.40 | .226 |
| Week 8 x Condition | -0.72 | 0.54 | -1.33 | -1.77, 0.34 | .185 |
| Week 16 x Condition | -0.70 | 0.56 | -1.25 | -1.78, 0.39 | .211 |
| Random Effects | | | | | |
| Intercept variance (σ^2^) | 2.61 | | | | |
| Residual variance (τ_00_) | 2.21 | | | | |
| N _participant_ | 79 | | | | |
| Observations | 245 | | | | |

*Note*. Values highlighted in bold indicate p-values that are statistically significant at an alpha level of .05. CI = confidence interval; SE = standard error.

**References**

1. Rinck M, Wiers RW, Becker ES, Lindenmeyer J. Relapse prevention in abstinent alcoholics by cognitive bias modification: Clinical effects of combining approach bias modification and attention bias modification. J Consult Clin Psychol. 2018;86(12):1005-16.

2. Wiers RW, Eberl C, Rinck M, Becker ES, Lindenmeyer J. Retraining automatic action tendencies changes alcoholic patients' approach bias for alcohol and improves treatment outcome. Psychol Sci. 2011;22(4):490-7.

3. Blackwell SE, Woud ML, MacLeod C. A Question of Control? Examining the Role of Control Conditions in Experimental Psychopathology using the Example of Cognitive Bias Modification Research. The Spanish Journal of Psychology. 2017;20:E54.

4. Kakoschke N, Kemps E, Tiggemann M. What is the appropriate control condition for approach bias modification? A response to commentary by Becker et al. (2017). Addictive Behaviors. 2018;77:295-6.
